# A Practical Guide to the Examination of the Eye

**Published:** 1898-12

**Authors:** 


**A Practical Guide to the Examination of the Eye.** By SIMEON SNELL. Pp. xiii., 177. Edinburgh: Young J. Pentland. 1898.

It can hardly be urged that there was an actual demand for yet another guide to the examination of the eye. But it must be readily admitted that the author has attained the special object he had in view, namely, that of providing a practical book which should be readily understood by the student and junior practitioner. There is much that is eminently to the point, and the descriptions of the various methods of examination are in most instances admirably clear; but yet we cannot help feeling that the book leaves a good deal to be desired.

The method of examination and the conditions, both normal and abnormal, to be found by it are most easily learnt concurrently; and where changes are likely to be overlooked, difficult to see, or of special diagnostic value, the attention should be particularly directed to the fact. In many cases this has been done, but the book decidedly errs by its numerous important omissions, and the special procedure and care necessary to secure the detection of some diseases are not sufficiently emphasised.

The illustrations are in most instances helpful, if not artistic; but some are superfluous, and those illustrating retinoscopy might well be replaced by diagrams explaining the principle on which the test is based.

In dealing with some subjects the author seems to have gone beyond the intended scope of the book. but the additional information is useful and on the whole accurate. There is, however, one glaring error which must be mentioned. The "angular method" for determining the angle of squint is entirely wrongly described. The patient is directed to fix *both* eyes on a distant point—which is impossible—whilst the position of the eye of the observer is not specified—which is absurd. Moreover the illustration (Fig. 85) is altogether misleading. How anyone familiar with the use of the method could pass such a description as the book gives is incomprehensible.

The writer acknowledges his indebtedness to various text-books, from which he quotes freely—upwards of ten pages are quoted *verbatim*. These passages are certainly well chosen, and it is convenient to have them collected in one volume. The reader will find many practical hints, and information on methods of investigation which are not sufficiently known. It cannot be said that the book is altogether fitted to replace already existing works on the examination of the eye, but there is much in it that makes it a useful supplement to them.

